# Unintentional Childhood Injuries in Urban and Rural Ujjain, India: A Community-Based Survey

**DOI:** 10.3390/children5020023

**Published:** 2018-02-08

**Authors:** Aditya Mathur, Love Mehra, Vishal Diwan, Ashish Pathak

**Affiliations:** 1 Department of Paediatrics, R. D. Gardi Medical College, Ujjain 456006, India; dr.adityamathur121@gmail.com (A.M.); lovemehra2000@gmail.com (L.M.); 2 Department of Public Health & Environment, R. D. Gardi Medical College, Ujjain 456006, India; vishaldiwan@hotmail.com; 3 Global Health—Health Systems and Policy, Department of Public Health Sciences, Karolinska Institutet, Stockholm SE-171 76, Sweden; 4 Department of Women and Children’s Health, International Maternal and Child Health Unit, Uppsala University, Uppsala SE-751 85, Sweden

**Keywords:** childhood injuries, incidence, epidemiology, risk factors, India

## Abstract

Injuries are a major global public health problem. There are very few community-based studies on childhood injury from India. The objective of this cross-sectional, community-based survey was to identify the incidence, type, and risk factors of unintentional childhood injuries. The study was done in seven villages and ten contiguous urban slums in Ujjain, India. World Health Organization (WHO) tested tools and definitions were used for the survey, which included 2518 households having 6308 children up to 18 years of age, with 2907 children from urban households and 3401 from rural households. The annual incidence of all injuries was 16.6%, 95% Confidence Interval 15.7–17.5%, (*n* = 1049). The incidence was significantly higher among boys compared to girls (20.2% versus 12.7%, respectively), was highest in age group 6–10 years of age (18.9%), and in urban locations (17.5%). The most commonly identified injury types were: physical injuries (71%), burns (16%), poisonings (10%), agriculture-related injuries (2%), near drowning (2%), and suffocations (2%). The most common place of injury was streets followed by home. The study identified incidence of different types of unintentional childhood injuries and factors associated with increased risk of unintentional injuries. The results can help in designing injury prevention strategies and awareness programs in similar settings.

## 1. Introduction

Injuries, especially unintentional injuries, are considered to be a leading cause of death among children and adolescents and a serious public health problem around the world [1,2]. Children are considered to be more vulnerable to unintentional injuries due to their curiosity to explore and experiment their surroundings, their small stature, and their physical and physiological immaturity to perceive danger [3,4,5]. Unintentional injuries among children are largely preventable causes of death and disability [6,7,8]. According to the World Health Organization (WHO)’s Global Burden of Disease Study estimates, unintentional injuries account for about 855,000 deaths in children and young people under the age of 18 years each year [9,10].

In addition, the burden of disability adds to the problem, as the burden of injuries are greater among children since they have more years ahead of them to be affected by disability [3,4,5,7,8,11]. The burden of injuries worldwide is excessively concentrated in low- and middle-income countries (LMIC) [4,5,9]. According to the WHO, more than 95% of all deaths from external causes among children occurred in the poorest countries [9]. Also, about 91% of unintentional injury deaths and 94% of disability-adjusted life-years (DALYs) were lost in LMIC in 2004 [2,9,11]. Unintentional injuries accounted for over 7% of total deaths and over 9% of total DALYs in these countries [2,9]. Childhood DALYs attributable to injury are highest in sub-Saharan Africa and India [2]. The Southeast Asian region contributes to about one-third of all global injuries [2]. However, there is little focus on injuries as a public health problem in South Asia and there is paucity of data on injuries from the region. Also, in India, 21.9% of the population lives below the national poverty line of 2 US dollars per day [12]. The province of Madhya Pradesh in India, where this study was done, has almost one-third of the population below the poverty line [12].

Most literature on childhood injuries from LMICs has been reported from hospital-based studies or trauma centers [13]. Hospital-based studies underestimate the true burden of injuries in LMICs, as many reportable injuries are not considered serious enough to visit a health care facility [14]. Hence, a community-based study on childhood injuries was done with the objective to study the incidence of childhood injuries in an urban and rural locality in Ujjain district in central India and also to determine the epidemiological risk factors associated with childhood injuries in the region.

## 2. Materials and Methods

### 2.1. Study Sites

The study was conducted by Department of Pediatrics, R.D. Gardi Medical College (RDGMC) in both urban and rural areas of Ujjain, a semi-rural district in the western Madhya Pradesh, India. Ujjain district has a population of 1.9 million within an area of 6091 km^2^ [15]. The population below age 15 years in urban and rural area is 26.4% and 30.4%, respectively [16]. The sex ratio of the total population (females per 1000 males) is 958 in urban and 975 in rural areas [16]. The proportion of the female population of age 6 years and above who have ever attended school is 76.4% in urban and 55.5% in rural areas [16]. The proportion of women who are literate in urban areas is 75.3%, while only 46.8% of women in rural are literate [16]. For rural sites, seven villages were randomly selected from Demographic Surveillance Sites (DSSs) of RDGMC [17]. The urban sites were ten geographically contiguous slums in Ujjain city, having 2000 households with 10,000 individuals around the urban health center of RDGMC.

### 2.2. Sample Size Calculation

Sample size was calculated based on the WHO guidelines for sample size calculation for community surveys of injuries [18]. For an assumed prevalence of 50% (r), a design effect of 2 (f), an estimated non-response rate of 10%, and an average household size of 5 (nh), p = 0.15, i.e., the proportion of children in population, the number of households that would need to be surveyed in order to estimate the prevalence of injuries in the whole population, with a margin of error of ±5%, is calculated as follows:

Number of households, n = [4 (r) (1 − r) (f) (1.1)]/[(e2) (p) (nh)](1)

where 4 = the factor to achieve 95% level of confidence r = 50%; 1.1 = factor necessary to raise the sample size by 10% to allow for non-responses; f = 2e = 5%; p = 1 (whole population); nh = 5,

n = [4 (0.50) (1 − 0.50) (2) (1.1)]/[(0.052) (0.15) (5)] n = 1173(2)

Thus, the minimum sample size for each site class (rural and urban) was 1173 children.

### 2.3. Sampling

For the rural study site, all the households in each of the selected villages were manually listed to obtain a sampling frame of households. A total of 1483 households were available to be surveyed from selected villages. For urban sites, each slum was divided in three clusters, each having a total of 47 to 50 households. Thus, a range of 1410 to 1500 households was available to be surveyed. In the case that a house did not have a child, the next nearest house was selected.

### 2.4. Data Collection Tools and Methods

Data collection was carried out during January 2017 to October 2017. All the households in the sampling frame were visited and household having children up to 18 years of age were approached to participate in the study. Written informed consent was obtained after explaining the purpose of the study. Three study assistants with previous household survey experience were trained over a two-week period for data collection. The female heads of the households were interviewed by one of the three trained study assistants. Two team leaders with fieldwork experience supervised the data collection. Data was collected using two questionnaires. The first structured questionnaire was used to collect socio-demographic information of the family head and household characteristics. The second questionnaire was developed using WHO TEACH-VIP 2 guidelines for surveys on injuries [14]. Only questions related to injury morbidity were included in the survey. The questionnaire was originally developed in English and then translated into Hindi by two experts in Hindi language [19]. Any discrepancy in the translation was resolved by consensus by an expert panel [19]. Then the questionnaire was again translated to English to ensure the original meaning of the questions had not changed and the questionnaire was then pilot tested on 50 respondents [19].

For the survey, injury was defined as (a) if the child was injured and treated with simple medical therapies by parents or another authorized adult; (b) if in a clinical setting the injury was diagnosed by a health care provider. The study assistants explained the WHO definition of an injury briefly and provided examples of external causes of injuries [14]. Any injury, as defined above, in the preceding 12 months of the survey was included. Data was collected on falls, road traffic injuries, burns, near drowning, poisonings, and suffocations. For the rural survey, questions related to agricultural injuries were also included. The questionnaire also gathered data on injury events, modes of injury, and the nature of each injury [14]. Any reported injury was included, regardless of whether medical care was given or the days of activity lost. A household with no occupants present was revisited, and if on subsequent visits no one was at home then the household was considered to be a non-response [14]. A revisit to household was done within one month of the first data collection to collect missing data, if any. Field supervisors reviewed the questionnaires daily, and 15% of the survey forms were rechecked for consistency and completion by the principal and co-investigators. The study was approved by the Institutional Ethics Committee of R.D. Gardi Medical College, Ujjain (Approval number-354/2014).

### 2.5. Data Management and Analysis

Data in the field was collected on paper forms. All entered information was then checked for completeness and consistency of responses manually. The data was coded, entered in Epi info 7 (7.1.3) CDC Atlanta, Georgia (USA). The analysis was performed using Stata (Version 12.0, StataCorp., College Station, TX, USA). Double entry verification was done on 5% of the data to check for and reduce data entry errors. Data was analyzed to determine the incidence and distribution of the injuries, socioeconomic characteristics of the injured children, types of injuries, and risk factors associated with the injuries. The differences between proportions were assessed using the *p*-value for heterogeneity. Chi-square and Fisher’s exact tests were used to estimate differences in variables. The crude odds ratio (OR) along with the associated 95% confidence intervals (CI) and *p* values were calculated from two-by-two tables. A *p*-value < 0.05 was considered statistically significant.

## 3. Results

### 3.1. Study Population

The survey included screening information from 16,004 individuals of all ages living in 2846 households. Within this sample there were 6308 children up to 18 years of age living in 2518 households. An average of 7441 and 8563 individuals lived in the urban and rural households, respectively. The survey included 2907 children from urban areas and 3401 from rural areas. The overall response rate was 98%. The non-response rate of 2% was due to households that were locked even after the follow-up visit.

### 3.2. Incidence of Unintentional Injuries

A total of 1049 children (16.6%, 95% CI 15.7 to 17.5) were reported to have had any form of injury in the previous one year. The annual incidence per 100 children of various unintentional injuries is presented in Table 1. The incidence of unintentional injuries was significantly higher in boys compared to girls, with girls having a 43% lower chance of having an injury (Table 2). The mean age of the injured children was 9.2 years (±0.15 years) (Standard Error). The highest incidence of injuries was in the 11 to 18 years age group, however, the differences were not statistically significant. Only the middle age group of 6–10 years had a higher risk of having any injury, as compared to younger children of up to 5 years or children between 11 to 18 years of age, with an OR of 1.21 (Table 1 and Table 2). Children from urban areas had slightly higher incidence then those from rural areas, but the difference was not statistically significant (Table 1 and Table 2).

### 3.3. Type and Mode of Injury

Physical injuries were the most frequent (*n* = 746/1049, 71%) type of injury, followed by burns (16%), and poisonings (10%), with agricultural injuries, near drowning, and suffocation injuries constituting nearly 2% each (Table 3).

The physical injuries included road traffic injuries (*n* = 229, 31%), fall injuries (*n* = 228, 31%), a child struck or hit (*n* = 263, 35%), and dog bites (*n* = 26, 3%). Burn injuries were mainly scald (n = 119, 70%) followed by fire-related injuries (*n* = 30, 18%) and electrocution (*n* = 21, 12%). Poisonings were more common in rural areas and were caused predominantly by insect bites (n = 52, 52%) and accidental ingestion of pesticides (*n* = 46, 46%). In our study, most (*n* = 22, 88%) cases of near drowning occurred in urban areas, and near drowning events were predominantly seen in boys. There was no statistically significant difference between age groups and risk of near drowning. Agriculture injuries were most common among boys (*n* = 13, 52%) and age group 11 to 18 years (*n* = 13, 52%). Suffocation injuries were more common among boys (*n* = 12, 57%) of urban areas (*n* = 12, 57%) and the most affected age group was 1 month to 5 years (*n* = 17, 81%). Except for burns, boys had higher incidence of all types of injuries compared to girls. Burns, poisonings, near drownings, and suffocation injuries were more common in younger children up to 5 years of age. However, physical injuries and agriculture-related injuries were more commonly seen in 11- to 18-year-olds compared to younger children. There was no statistically significant difference in physical injuries in rural and urban areas. However, poisonings and agriculture-related injuries were more common in rural areas and burns, near drowning, and suffocation injuries were more common in urban areas. Among children that were eligible to go to school, most (52%) were in elementary school. A total of 13% children did some kind of paid or unpaid work.

### 3.4. Place of Injury

The two most common places of injuries were home and road for the majority of injuries except for agriculture-related injuries and near drowning. The details are shown in Table 4.

## 4. Discussion

To our knowledge, this is the first community-based survey of childhood injuries from central India. The incidence of unintentional injuries in urban and rural community in children up to 18 years of age in the present study was 16.6%. The overall incidence of injuries was higher in boys compared to girls and was highest in the age group of 6 to 10 years compared to younger children. Injuries due to road traffic accidents and falls were the most common type of injury. There were notable differences in the injury patterns in the rural and urban areas, with poisonings and agriculture-related injuries seen more commonly in rural areas and burns, near drowning, and suffocation injuries were more common in urban areas.

A study of urban slums in the Delhi area of India reported an incidence of 7.1% and included children up to 18 years of age [20]. Another Indian study reported an incidence of 11% [21]. Other community-based studies from India have reported variable incidence between 6% and 23% [20,21,22,23,24]. The high variability in the incidence of injuries is due to differences in the age of the populations of children included in the studies, and also due to variable definition of injuries used in the studies. The incidence in the present study is higher than the annual incidence rate of 24.6/1000 population reported from Nepal [25] but is lower than that reported from Bangladesh [26].

Our study showed a significantly higher injury incidence among boys compared to girls (20.2% versus 12.7%, respectively). Similar patterns have been reported from previous studies in India [20,21,22,24], Nepal [25], Bangladesh [26], Pakistan [27,28], Saudi Arabia [29], Ghana [30], and Colombia [31]. Boys are injured more frequently due to the patriarchal nature of societies in many LMICs including India, where boys are allowed autonomy to explore their environment at an early age [32].

Among burn injuries, however, girls had higher incidence compared to boys in our study. A review of burn injury studies from South Asia showed a similar pattern of trend reversal, with girls being more affected than boys beyond one year of age [33]. The pattern of burn injuries can be explained by deep-rooted gender-based roles for girls in LMICs including India, which exposes them to unsafe kitchen or cooking areas [32]. Girls also have increased risk of fire-related injuries in the cooking areas from cooking utensils, which are not designed for handling by children with limited capacity [24].

The distribution of the types of injuries identified in our study was similar to unintentional childhood injuries reported by the WHO in LMICs [9,10]. However, low socio-economic status is not detrimental in all settings and in all types of injuries, both within a country and between countries [34,35]. Therefore, socio-economic status alone cannot explain the high concentration of injuries in LMICs. Globalization and its economic and socio-cultural effects have been viewed as one of the contributing factors in increasing the risk of unintentional injuries in LMICs [34].

Worldwide, burns are a significant cause of injury-related morbidity and mortality, and South Asian countries including India bear a significant burden of injuries due to burn [33]. In India, the incidence of burn injury varies between hundred thousand to 2 million, annually [33]. In our study, children between ages 1 month to 5 years had the highest incidence of burn injuries and burn injuries were more common in girls. Poisoning is the fourth highest cause of deaths and DALYs in high-income countries, while it accounts for a smaller proportion of unintentional injuries in LMICs [1,11]. The incidence of poisonings in our study was higher than that reported by a study done in four cities from LMICs [36] and a study from Wardha, India [22]. This may be due to population mix or urban and rural children in our study, as poisonings are more common in rural areas and most were due to insect bites and pesticide ingestion [5,11,22,37]. In our study, the boys were more at risk for poisonings, which is similar to the findings of other studies [5,11,22,37]. In the present study, more cases of near drowning occurred among boys and majority of near drowning occurred in urban areas. Almost all age groups were equally at risk for near drowning. These findings were unlike those previously reported from India [37]. The reason might be that the study sites in the rural areas did not have water bodies nearby, while the urban sites were close to water bodies such as river “*Kshipra*” and big runnels, as both of these passes through the city. Suffocation injuries were most common among the 1 month to 5 years age group, with male predominance and homes representing the most common places of injury. These findings are consistent with a previous World Report on Child Injury Prevention by the WHO [9]. In our study, agriculture injuries were more common among boys and the most affected age group was 11 to 18 years. These findings are consistent with a previous study from India [24]. In our study, the majority of the injuries occurred on roads followed by home. These findings are in line with other studies [4,6,9,11,34,37].

### 4.1. Recommendations

On the basis of our results, we provide the following suggestions for the local, state, and central level governments, as well as schools and families, in order to develop effective measures to control the incidence of injuries. The governments should focus and invest more resources on injury control and prevention. There is a need to implement a comprehensive and appropriate strategy for road safety. There is also a need to educate parents of children, and pediatricians can play a significant role in this [3]. Educating children and adolescents about safety and necessary precautions for preventing injuries can be effective in reducing the incidence of injuries. Thus, injury prevention should be part of the school curriculum [38,39]. This study also recommends modification of the home environment for the reduction of injuries, home safety education, and the provision of safety equipment for the prevention of injuries [4]. Finally, the local government and schools should collaborate in establishing an injury surveillance system to provide theoretical and empirical support for the injury prevention program.

### 4.2. Methodological Considerations

Our study had the following limitations: firstly, this being a cross-sectional study, we cannot draw clear conclusions about causality; secondly, an under-reporting due to recall bias might have occurred, as information on the incidence of injuries was collected retrospectively, however, this limitation is natural in all community-based surveys. We have not presented all the details of physical injuries, poisonings, and burn injuries, as we would like to present them as separate studies. The major strengths of our study are: our study provides much needed data at the community level; we used standard, validated injury survey tools recommended by the WHO after pilot testing; and local and experienced research assistants carried out the survey using local language “*maalvi*”, which resulted in high response rates.

## 5. Conclusions

This community-based study provided a snapshot of injury burden by identifying the incidences of different types of injuries in Ujjain, India. The study highlighted various age, sex, and socio-demographic risk factors associated with injury. Our study identified homes and roads/streets as the most common places for the occurrences of injury in children and adolescents.

## Figures and Tables

**Table 1:** Annual incidence of unintentional injuries in Ujjain.

Variable	Surveyed Children (*n* = 6308)	Children Identified with any Unintentional Injury	Annual Incidence of Unintentional Injury per 100 Children (95% CI)	*p-*Value
**Sex**				
Boys	3252	658	20.2 (18.8–21.6)	<0.001
Girls	3056	391	12.7 (11.6–13.9)	
**Age group**				
1 month–5 year	1926	312	16.1 (14.5–17.8)	Reference
6–10 years	1680	319	18.9 (17.1–20.8)	0.028
11–18 years	2702	418	15.4 (14.1–16.8)	0.502
**Location**				
Rural	3401	540	15.8 (14.6–17.1)	0.083
Urban	2907	509	17.5 (16.1–18.8)	

CI-confidence interval.

**Table 2:** Risk of unintentional injury according to sex, age categories, and rural or urban locality of residence of the injured children (*n* = 1049) included in the study.

	Total (%) *	Unintentional Injury		OR	95 CI	*p*-Value
	6308	No (%) ^#^ 5259	Yes (%) ^#^ 1049			
**Sex**						
Boys	3252 (52)	2594 (80)	658 (20)	0.57	0.50–0.66	<0.001
Girls	3056 (48)	2665 (87)	391 (13)			
**Age category**						
1 day–5 year	1926 (30)	1614 (84)	312 (16)	Reference		
6 to 10 years	1680 (27)	1361 (81)	319 (19)	1.21	1.02–1.43	0.028
11 to 18 years	2702 (43)	2284 (85)	418 (15)	0.94	0.80–1.11	0.502
**Location**						
Rural	3401 (54)	2861 (84)	540 (16)	1.12	0.98–1.28	0.083
Urban	2907 (46)	2398 (82)	509 (18)			

* Column percentage, ^#^ Row percentage, OR-Odds Ratio, CI-confidence interval.

**Table 3:** Distribution of type of injury by sex, age categories, rural or urban location, education, and occupation of the injured children (*n* = 1049) included in the study.

Variable	Total (%) * 1049	Physical Injuries ^^^ 746 (71)	Burns 170 (16)	Poisonings 100 (10)	Agricultural Injuries 25 (2)	Near Drowning 25 (2)	Suffocations 21 (2)
**Sex**							
Boys	658 (63)	498 (66)	80 (47)	62 (62)	13 (52)	14 (56)	12 (57)
Girls	391 (37)	248 (33)	90 (53)	38 (38)	12 (48)	11 (44)	9 (43)
**Age Category**							
1 month–5 years *n* (%) ^#^	312 (30)	189 (25)	70 (41)	52 (52)	0 (0)	9 (36)	17 (81)
6–10 years *n* (%) ^#^	319 (30)	247 (33)	38 (22)	20 (20)	12 (48)	7 (28)	4 (19)
11–18 years *n* (%) ^#^	418 (40)	310 (42)	62 (37)	28 (28)	13 (52)	9(36)	0 (0)
**Location**							
Rural	540 (51)	369 (49)	68 (40)	73 (73)	24 (96)	3 (12)	9 (43)
Urban	509 (49)	377 (51)	102 (60)	27 (27)	1 (4)	22 (88)	12 (57)
**Education status of injured**							
Not eligible for school *n* (%) ^#^	212 (20)	111 (15)	61 (36)	45 (45)	0 (0)	5 (20)	10 (48)
Uneducated *n* (%) ^#^	73 (7)	48 (6)	8 (5)	6 (6)	8 (32)	2 (8)	1 (4)
Elementary school *n* (%) ^#^	548 (52)	422 (57)	72 (42)	34 (34)	12 (48)	13 (52)	10 (48)
High school–high secondary school *n* (%) ^#^	216 (21)	165 (22)	29 (17)	15 (15)	5 (20)	5 (20)	0 (0)
**Occupation of injured**							
NA ^†^ *n* (%) ^#^	212 (20)	111 (15)	61 (36)	45 (45)	0 (0)	5 (20)	10 (48)
Paid work *n* (%) ^#^	72 (7)	59 (8)	8 (5)	3 (3)	2 (8)	1 (4)	0 (0)
Non-paid worker *n* (%) ^#^	59 (6)	42 (6)	9 (5)	6 (6)	1 (4)	1 (4)	1 (4)
Student *n* (%) ^#^	706 (67)	534 (71)	92 (54)	46 (40)	22 (88)	18 (72)	10 (48)

^^^ Physical injury covered road traffic injuries (*n* = 229), fall injuries (*n* = 228), child struck/hit (*n* = 263), and dog bites (*n* = 26). ^#^ Row percentage, * Column percentage, ^†^ Child less than 4 years old.

**Table 4:** Distribution of type of injury by the place of occurrence of the injured children (*n* = 1049) included in the study.

	Physical Injury ^^^ *n* (%)	Burns *n* (%)	Poisonings *n* (%)	Agricultural Injury *n* (%)	Near Drownings *n* (%)	Suffocations *n* (%)
Home	249 (33)	149 (87)	51 (51)	5 (20)	-	21 (100)
School	22 (3)	-	-	-	-	-
Road/street	392 (53)	15 (9)	12 (12)	-	-	-
Work place	25 (3)	5 (3)	1 (1)	-	-	-
Farm/agriculture area	11 (1)	1 (1)	11 (11)	20 (80)	-	-
Sports area	42 (6)	-	25 (25)	-	-	-
Canal/river/pond	5 (1)	-	-	-	25 (100)	-
Total	746 (100)	170 (100)	100 (100)	25 (100)	25 (100)	21 (100)

^^^ Physical injury covered road traffic injuries (*n* = 229), fall injuries (*n* = 228), child struck/hit (*n* = 263), and dog bites (*n* = 26).
